# Supplementary material for: Tbx3-dependent amplifying stem cell progeny drives interfollicular epidermal expansion during pregnancy and regeneration
Source: Nat Commun. 2017 Sep 11;8:508. doi: 10.1038/s41467-017-00433-7 (PMC5593911; doi:10.1038/s41467-017-00433-7)
Supplement: Supplementary file 2 — Supplementary Information [file 41467_2017_433_MOESM2_ESM.pdf]

Title: Supplementary Information

Description: Supplementary Figures

Title: Peer Review File

Description:

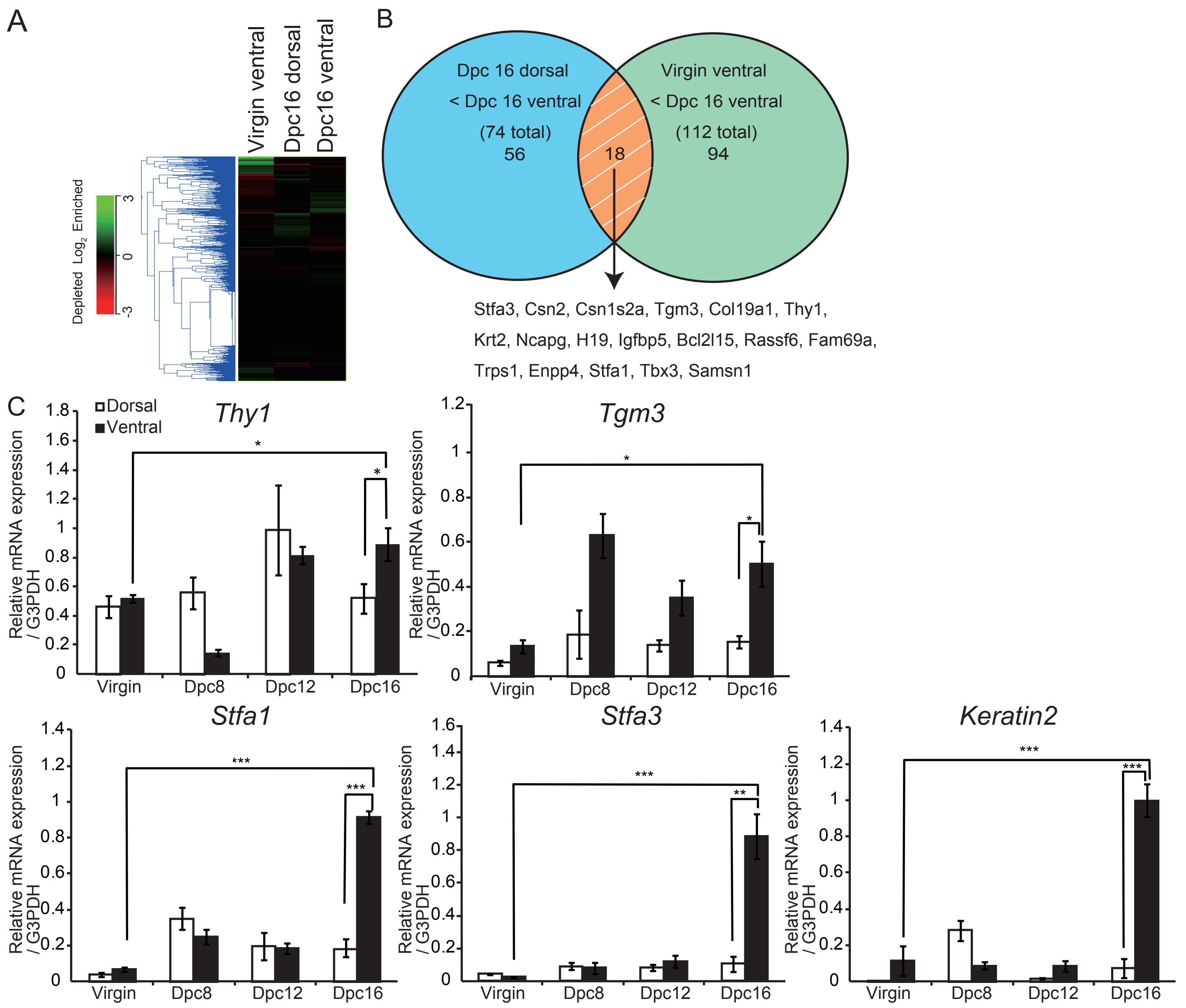

Supplementary Figure 1. Analysis of gene expression profiles of abdominal IFE basal cells in pregnant mice.

(A) Unsupervised hierarchical clustering and heat map display of genes differentially expressed in FACS-purified basal cells from ventral skin of virgin mice, ventral skin of 16 dpc mice, and dorsal skin of 16 dpc mice.

(B) Venn diagram showing the overlap between genes upregulated (>2 fold) in ventral skin of 16 dpc mice compared with dorsal skin of 16 dpc mice and ventral skin of virgin mice. (C) qPCR analysis of the indicated genes in dorsal and ventral skin of virgin and 8–16 dpc mice. Data represent the mean ± sem. (n=3 mice); *P<0.05, ***P<0.001, analysed by Dunnett’ s multiple comparison test.

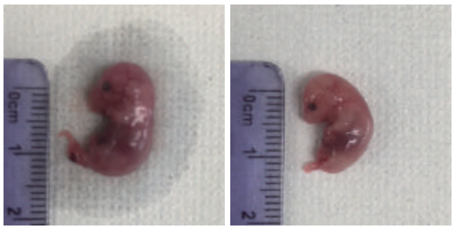

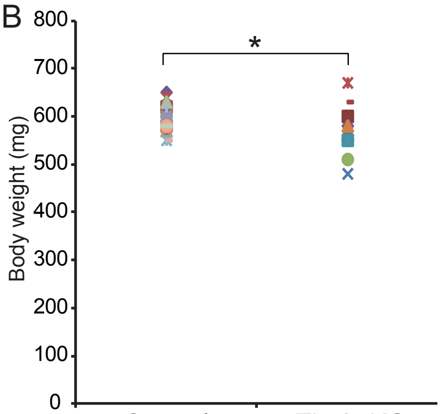

Supplementary Figure 2. *Tbx3* deficiency in maternal abdominal skin partially decreases the fetal body weight.
(A) Control (*Tbx3$^{flox/flox}$/WT*) or *Tbx3* cKO (*Tbx3$^{flox/flox}$/K14-CreER*) female mice were mated with genetically unaltered wild-type male mice. Tamoxifen (1 mg) was topically applied to abdominal skin of mother mice every day at 8–15 dpc. Gross appearances of embryos from 16 dpc control and *Tbx3* cKO mother mice are shown. Experiments were repeated three times with three mother mice for representative images. (B) Body weights of embryos from control and *Tbx3* cKO mothers. Data represent the mean ± sem. (n=27 and 15 embryos pooled from three control and *Tbx3* cKO mother mice, respectively); *P<0.05, analysed by the two-tailed t-test.

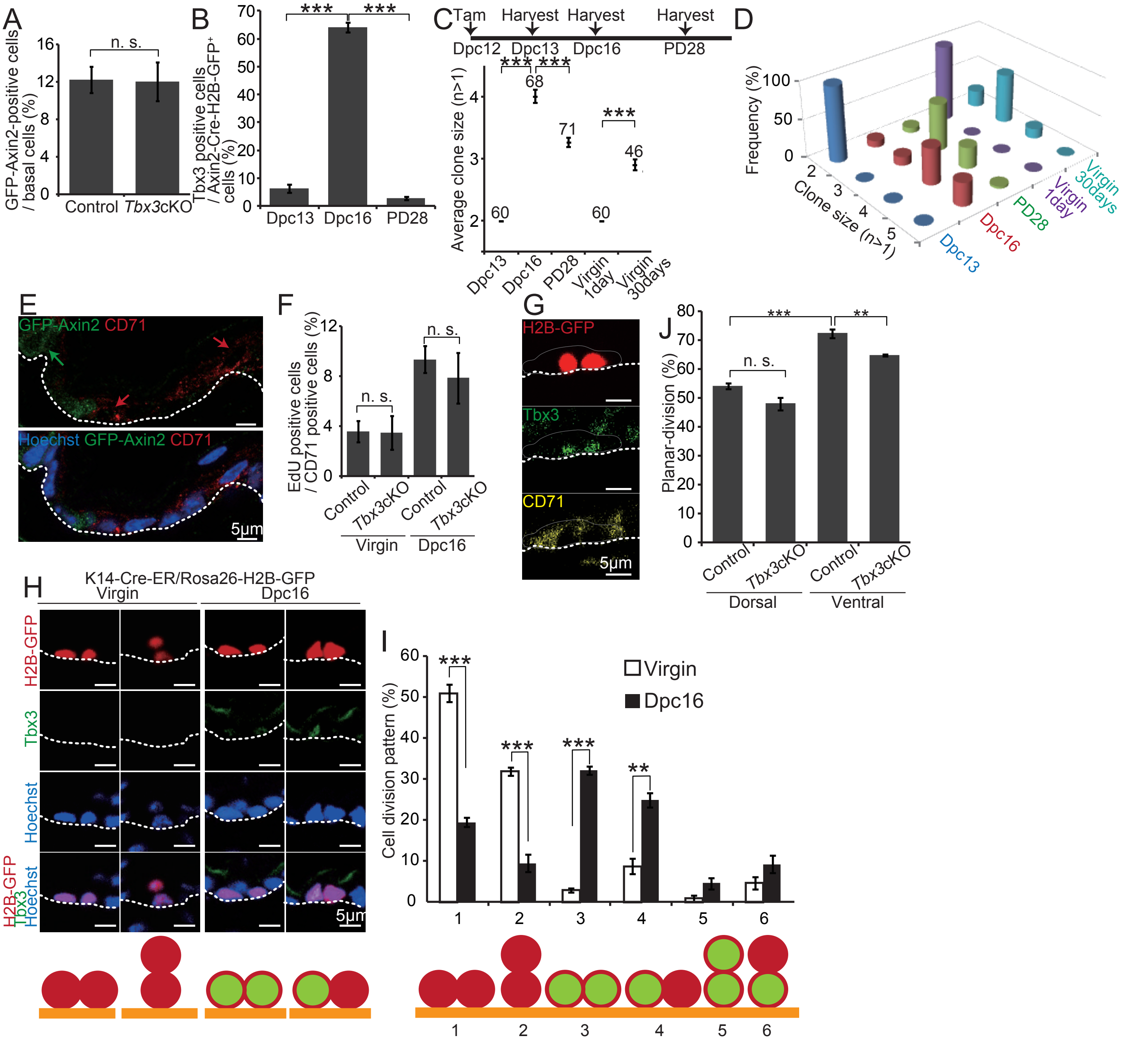

Supplementary Figure 3

Supplementary Figure 3: Characterisation of Axin2$^+$ and Tbx3$^+$ IFE basal cells in abdominal skin of pregnant mice.
(A) Quantification of Axin2-d2EGFP$^+$ cells in IFE basal layers of ventral skin from *Axin2-d2EGFP* virgin mice (Control) and *Axin2-d2EGFP/Tbx3* cKO virgin mice. To induce Tbx3 deletion, tamoxifen (1 mg) was topically applied to abdominal skin once a day for 5 days, and the skin tissues were harvested at 21 days after tamoxifen administration. (B–D) *Axin2-Cre-ER/Rosa26-H2B-GFP* mice were injected with tamoxifen (3 mg/25 g body weight) at 12 dpc, and skin tissues were harvested at 13 dpc, 16 dpc, and PD28. (B) Quantification of Tbx3$^+$ cells among Axin2-Cre-ER-labelled cells in IFE basal layers. (C and D) Average size (C) and size distribution (D) of Axin2-Cre-ER-labelled clones as measured by the basal cell number per clone in each section. For the control, skin tissues were obtained from virgin mice at 1 or 30 days after tamoxifen administration. The number of analysed clones pooled from three mice is indicated in C. Data represent the mean ±sem.; ***P<0.001 analysed by Tukey' s multiple comparison test. (A, B) Data represent the mean ±sem. of averages of three independent experiments (n=3). >500 cells(A) and >150 cells (B) were analyzed to calculate the average in each experiment; ***P<0.001, analysed by the two-tailed t-test. (E) Sections of ventral skin from 16 dpc *Axin2-d2EGFP* mice immunostained with anti-GFP and anti-CD71 antibodies, and counterstained with Hoechst. Experiments were repeated three times with three mice for representative images. (F) Quantification of EdU$^+$ cells in the CD71$^+$ cell population in IFE basal layers of ventral skin from virgin or 16 dpc control and *Tbx3* cKO mice. Data represent the mean ±sem. of averages of three independent experiments (n=3), analysed by Tukey' s multiple comparison test. >500 cells were analyzed to calculate the average in each experiment. (G) *Axin2-Cre-ER/Rosa26-H2B-GFP* mice were treated as described in Fig. 3m, immunostained with anti-GFP, anti-Tbx3, and anti-CD71 antibodies, and counterstained with Hoechst. Typical images of symmetric division of Axin2-Cre-ER-labelled basal cells are shown. (H) Sections of ventral skin from virgin and 16 dpc *K14-Cre-ER/Rosa26-H2B-GFP* mice immunostained with anti-GFP and anti-Tbx3 antibodies, and counterstained with Hoechst. Tamoxifen (0.2 mg/25 g body weight) was injected at 12 dpc. (I) Quantification of the division pattern of K14-Cre-ER-labelled IFE basal cells in ventral skin. Data represent the mean ±sem. of averages of three independent experiments (n=3). >50 labelled cells (virgin) or n>70 labelled cells (Dpc 16) were analyzed to calculate the average in each experiment; **P<0.01, ***P<0.001 analysed by Tukey' s multiple comparison test. (J) Quantification of planar-oriented division of IFE basal cells in dorsal and ventral skin from 16 dpc control mice (*Tbx3$^{flox/flox}$/WT*) and 16 dpc *Tbx3* cKO (*Tbx3$^{flox/flox}$/K14-CreER*) mice following tamoxifen administration at 12 dpc. Planar-oriented cell division was determined based on the staining patterns of survivin (see Fig. 3m). Data represent the mean ±sem. of averages of three independent experiments (n=3). >60 survivin$^+$ cells were analyzed to calculate the average in each experiment; **P<0.01, ***P<0.001 analysed by Dunnett' s multiple comparison test.

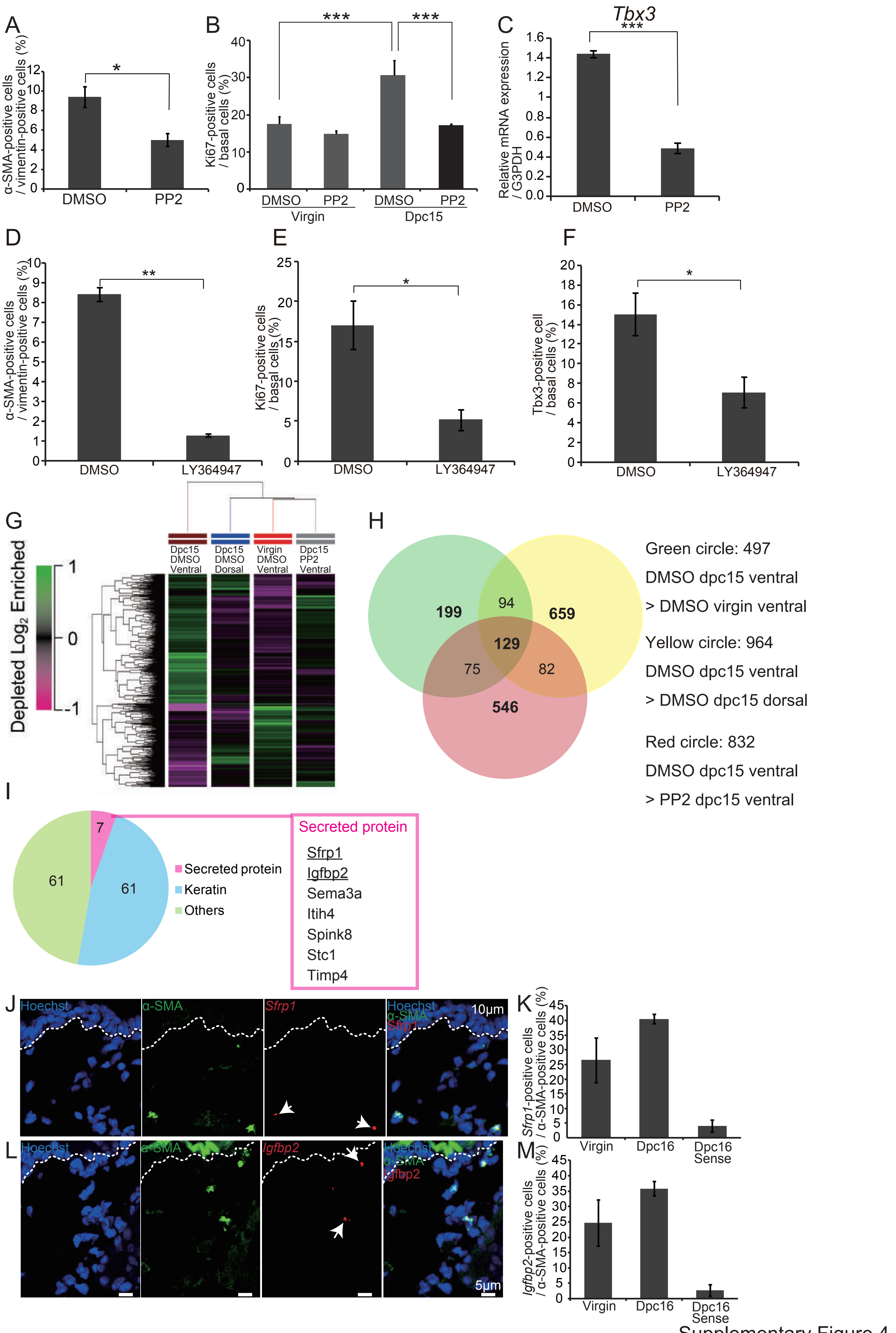

Supplementary Figure 4

Supplementary Figure 4: Characterisation of α-SMA$^+$/vimentin$^+$ dermal cells in abdominal skin of pregnant mice.

(A–C) Quantification of α-SMA$^+$ cells in the vimentin$^+$ dermal cell population (A), Ki67$^+$ IFE basal cells (B), and *Tbx3* mRNA expression levels in whole skin tissues (C) in the ventral skin of 16 dpc mice injected with DMSO or PP2.

Data represent the mean ±sem. of averages of three independent experiments (n=3). >100 cells (A) or n>500 cells (B) were analyzed to calculate the average in each experiment; *P<0.05, ***P<0.001, analysed by Dunnett' s multiple comparison test (B) or the two-tailed t-test (A and C). (D–F) Quantification of α-SMA$^+$ cells in the vimentin$^+$ dermal cell population (D), Ki67$^+$ IFE basal cells (E), and Tbx3$^+$ IFE basal cells (F) in ventral skin of 16 dpc mice injected with DMSO or LY364947. Data represent the mean ±sem. of averages of three independent experiments (n=3). >100 cells (D) or n>500 (E and F) cells were analyzed to calculate the average in each experiment; *P<0.05, **P<0.01, analysed by the two-tailed t-test. (G) Unsupervised hierarchical clustering and heat map display of genes differentially expressed in the dermis from ventral skin of virgin mice, ventral skin of 15 dpc mice, dorsal skin of 15 dpc mice, and ventral skin of 15 dpc mice injected with PP2. (H) Venn diagram showing the overlap between genes upregulated (>2 fold) in the dermis from ventral skin of 15 dpc mice compared with ventral skin of virgin mice, dorsal skin of 15 dpc mice, and ventral skin of 15 dpc mice injected with PP2. (I) List of genes encoding secretory proteins upregulated in the dermis from ventral skin of 15 dpc mice. (J and L) FISH analysis of *Sfrp1* (J) and *Igfbp2* (L) mRNAs using anti-sense probes in combination with immunostaining using an anti-α-SMA antibody and Hoechst staining in ventral skin of 16 dpc mice. (K and M) Quantification of α-SMA$^+$ dermal cells expressing *Sfrp1* (K) and *Igfbp2* (M). Data represent the mean ±sem. of averages of three independent experiments (n=3). >100 cells were analyzed to calculate the average in each experiment; analysed by Dunnett' s multiple comparison test.

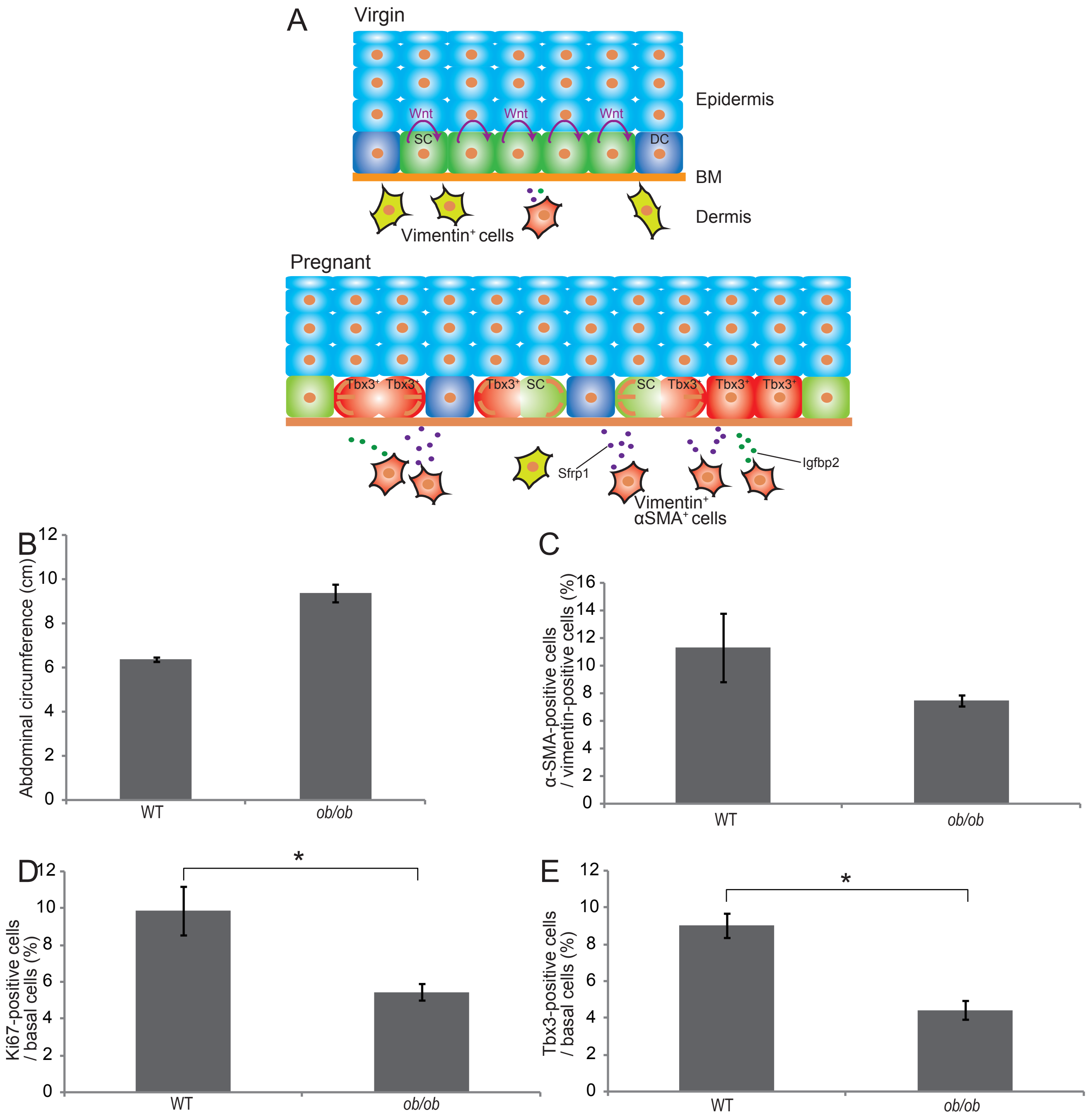

Supplementary Figure 5: Difference in cell dynamicity between abdominal skin of pregnant mice and obese mice.

(A) Schematic diagram of IFE basal cell dynamicity in abdominal skin of virgin and pregnant mice.

BM, basement membrane; SC, stem cell; $Tbx3^+$, Tbx3-positive cell; DC, differentiated cell.

(B) Abdominal circumferences of wild-type and *ob/ob* mice. Data represent the mean ± sem. (n=2 mice).

(C–E) Quantification of α-SMA^+ cells in the $vimentin^+$ dermal cell population (C), $Ki67^+$ IFE basal cells (D), and $Tbx3^+$ IFE basal cells (E) in ventral skin of wild-type and *ob/ob* mice.

Data represent the mean ±sem. of averages of three independent experiments (n=3). >100 cells (C), n>500 cells (D and E) were analyzed to calculate the average in each experiment; *P<0.05, analysed by the two-tailed t-test.
